# Reports on Hospitals of the United Kingdom

**Published:** 1914-02-14

**Authors:** Henry Burdett


### REPORTS ON
### Hospitals of the United Kingdom.

By SIR HENRY BURDETT, K.C.B., K.C.V.O.

SERIES III.

### THE NORTHAMPTON GENERAL HOSPITAL.

This hospital was established in 1743. It continued its work in the same buildings, with certain additions, for some 160 years. Then a warm controversy arose as to whether the existing buildings could be reconstructed or what was the proper course to pursue. The hospital had at that time been subject to epidemic outbreaks of disease. Experts were consulted, and they connected them with the deposits of material contained in the ventilators and in the walls of the wards, which had been colourwashed for a long series of years until the accumulation of the coats, one on top of the other, attained a thickness of several inches. A great accumulation of débris was found beneath the floors, and from these materials Professor Sims Woodhead, F.R.S., made a number of cultures which contained micrococci representing various diseases. In the result the governors determined to adopt a scheme whereby new blocks of wards were erected in 1903-4 behind the old buildings, and the latter were converted into an administration block. The result has been to give the county of Northampton an up-to-date hospital, which has been made more complete during the last two years by the erection of a new laundry, the enlarging and remodelling of the mortuary unit, and by additions to the outpatients' department. A new electrical branch and the full equipment of the *x*-ray department have recently been completed. These latter have been excellently well done, showing that Dr. Robson, the member of the staff responsible for them, has a thorough and unusual knowledge of this important section of hospital work. The hospital is at the present time in urgent need of a Radium Fund. There can be no doubt that if the Northampton General Hospital had such a fund a great deal of good could be done.

### The Call for Men of Energy.

Like other county towns Northampton exhibits, if we may judge from the support given to the hospital now and thirty years ago, a want of appreciation and knowledge of the great additional cost of running a modern up-to-date hospital. This fact is bad enough in itself, but it becomes infinitely worse when it is accompanied by the failure to grasp, the enormous value of the present General Hospital to Northampton in its up-to-date and perfect organisation. In the quality of the skill and knowledge brought to bear in every department of its work, this hospital will compare favourably with many of its type. It is not a little remarkable that in a manufacturing town, where the population has grown in thirty years by thousands, where the manufacturers have increased and prospered greatly, there should not have been a spontaneous recognition of the great developments which have characterised the treatment at this hospital, and the number of patients admitted and cared for in its wards. These developments should have been spontaneously met by the addition of at least £2,000 a year to its income. This sum is required to maintain the work at its present high efficiency. We heard, during our stay in Northampton, that the infirmary was always asking for money. So far from this fact, if it be true, reflecting on the managers of the infirmary or its medical staff, it really condemns the inhabitants of Northampton who have taken so little part in the support of this hospital, to which they owe a deep debt of gratitude. If we could have taken every individual of Northampton with us during our inspection; if we could have caused them to see, as we saw, the actual quality of the work and its paramount value to the patients and their dependants directly, and indirectly to every resident in the town and county of Northampton, who may at any time need skilled aid and care in the day of sickness, Monday morning would have seen hundreds of Northamptonians becoming subscribers. We wish to say quite frankly, and we have had some personal experience of what the men and women of Northampton can do and will do if properly approached, in the way of contributions to the hospital, that the present condition of its finances reflects seriously upon those at the head of its financial administration. Some steps should be taken to find a few men of energy and driving force who will not be content to quibble over every small item of necessary expenditure, but who are capable of taking a larger view of what the actual needs of the moment may be, to supply these needs, and then to make and keep the General Hospital prosperous and efficient.

### Pay the Sisters and Nurses.

Someone should secure that everyone employed within its walls shall have adequate remuneration. It is hardly credible, but it is a fact, that the remuneration offered to the sisters in the wards commences at £28 per annum, a sum at which it has stood for very many years. No other hospital of its size and reputation that we have discovered offers its sisters less than £30, and most of them give £35 rising to £40 per annum. Further, in the case of the better administered institutions, the sister's salary commences at a higher sum and increases up to £45 a year. Again, we observed that the private nursing staff earned profits amounting to £177 for the year ending July, 1913, but no mention is given of the proportion of these profits which have been given in bonuses to the nurses who earned them. Guy's

Hospital has one of the fairest and best schemes for remunerating the private nursing staff, and we would suggest that particulars of it should be obtained, and that the committee at Northampton should adopt a similar plan in dealing with the private nursing staff of the General Hospital. We are sure the committee will agree it would never do to run the risk of having the General Hospital, Northampton, included amongst those institutions which sweat their nurses.

### Some Shocking Lavatories.

Another thing we observed with regret was the shocking conditions, from a moral and an actual point of view, of the accommodation provided for the wardmaids and the domestic staff as to lavatories. Quite half the accommodation for the female staff presents an air of desolation and neglect that is really shocking, for in one lavatory three out of six basins were either broken or absent. It is not right for an institution of the importance of this hospital to provide for its maids nothing but a row of lavatory basins without the slightest provision for privacy and decency as we found to be the case. It would seem as if no one at present pays regular visits of inspection to this part of the hospital, or makes it a practice to inspect thoroughly all of the nurses' rooms and the condition of everything connected with them, at least once a month. We hope that steps will be taken to remedy the grave defects just alluded to, and to provide for an adequate and regular inspection of all the sleeping accommodation throughout the hospital.

### The Nurses' Quarters.

The nurses' quarters were, on the whole, in a much better condition than when we last inspected them, but the sisters ought to have a better and larger off-duty room than that at present allotted to them. We satisfied ourselves that, either by throwing an adjacent room into the present sisters' off-duty room, or by converting space available elsewhere, a suitable apartment can be readily found and provided at relatively little cost.

### The Kitchen and Stores Department.

We spent considerable time in these departments, which throughout present a smart appearance, and when taken in detail were found to exhibit continuous and conscientious labour and no little practical knowledge in various forms. We were much impressed with the excellent condition of these basement rooms, which reflect great credit on the housekeeper, the cook, and all who are responsible for their condition. There is no part more creditable to the workers of this hospital than the kitchen and stores department in the basement as we found it on the day of our visit.

### Some Matters which should be Changed.

The system on which coals are handled and distributed to the wards and other apartments probably dates from the earliest days. It is so out of date and so rough and ready as to be quite remarkable. The plan is to have a big, unsightly wooden receptacle on each floor, occupying valuable space in a large room that would often make a good ward, where the coal is distributed. The idea is that the dirt is shut up in these apartments, for there are several of them; but we deny the justice of this claim, for in fact it perpetuates the distribution of coal dust on the floors of the hospital. All the filling of hods and trolleys for the supply of coal should be done in the basement, and this detail in distribution deserves the immediate attention of the committee, who should abolish the old and archaic system to which we refer. It is attended by another and most serious evil which is cruel and unnecessary. Cruel because when a patient has undergone a severe operation, or is acutely and seriously ill, noise is one of the most trying and painful experiences life provides. To-day the patients in the private wards, the patients in the general wards, and the medical staff complain bitterly of the present noisy system of distributing the coal. Such trolleys as there are are not suitable nor provided with wheels or tyres which would enable a silent distribution of the coal such as prevails in the best administered hospitals. What we suggest is that a small committee should be appointed to collect information and obtain drawings of the coal waggons and the way the coal is distributed in some of the best administered hospitals, that this committee should go into the whole question and the waste of space represented by the coal rooms, look thoroughly into the lifts accommodation of the hospital, and ensure that the whole of this minor, but in some senses important, detail of domestic management shall be brought up to date.

### Some Recent Additions.

Taking the new additions in order, the first is the laundry. This is now an ample, spacious, and, as far as machinery is concerned, well-equipped unit, but it fails in the hospital sense because those who were responsible for the plans seemed to have sadly lacked practical knowledge of the requirements of a hospital laundry. It has only recently been opened, and yet it contains no sorting room for dirty linen, and no separate accommodation for the reception and treatment of foul and septic linen. These two faults will have to be remedied, meaning further building at more cost. All this should have been avoided before the plans were finally approved.

### The Mortuary.

Every department of the mortuary has now been brought well up to date, and this unit is at present a credit to the institution. The new view room, which, we believe, was added mainly through the representations of the Matron, reflects great credit on her, and cannot fail to be an asset to the hospital by inculcating reverence in the treatment of the dead. This rest room or chapel contains no word of hope, in fact no word at all. However, seeing that we do not live in a pagan country, we think the point has only to be mentioned to secure a prompt remedy for this oversight. We know the curse of sects makes caution necessary, but in a

Christian country there should be no objection to: "I am the Resurrection and the Life," or "Till the day breaks, and the shadows flee away." In some hospitals we have seen the word "Asleep" over the entrance to the mortuary chapel. This word strikes the beholder and has been found helpful to many. The additions to the out-patient department, and the excellent electrical and *x*-ray installations, have been already mentioned.

### WHAT THE HOSPITAL NEEDS.

The facts that there is such an overflowing attendance at the out-patients' department, and that there is a growing increase in the number of in-patients, seem to warrant an inquiry by the committee into the number of insured persons treated as out-patients, to ascertain whether their treatment does not properly devolve upon their panel doctors rather than upon the hospital authorities. In any case, there is urgent need for the appointment of a sister to look after the out-patient work, as there is also the need for a small theatre in connection with the out-patient department. At the present time the sister to the operation theatre has, we understand, to share the out-patient work, and the out-patients needing operations are put into the operation theatre! Such a practice is wrong, hygienically and administratively. Hygienically, because it might have serious results to the in-patients for various reasons; administratively, because the actual volume of work done in the out-patient department at the present time necessitates the employment of a special sister to attend to it.

### "WE HAVE NO MONEY!"

We are well aware that the suggestions and criticisms just made may be met, as every suggestion is met by certain people, with the plea that there is no money. In reply, may we ask, "Whose fault is that?" There is an abundance of money in Northampton and district, providing sufficient energy is brought to bear on appealing for it. Our answer, then, must be that the reason that there is not enough money is that the committee, or those especially responsible for the financial work of the hospital, have not fulfilled their responsibility adequately. It must be manifest to many members of the committee, who are men of business, and for whom we have the greatest respect, that where a man does not secure the necessary results, it is essential to strengthen him or to find someone else to undertake the work. Many hospitals make it a rule to change the chairman of their financial committee periodically. Five years of serious service, where the duties are energetically fulfilled, are usually enough for any man to render in voluntary services to the hospital in the district in which he resides. The Northampton County Hospital needs an immediate addition to its income of £2,000 a year. This money can be obtained, and ought to be obtained, within the next six months, if the proper means are adopted and the right men are found to take the lead in this work. The hospital receives a contribution from the League of Mercy, and the principle of personal service in days of health in the cause of the sick, which that League represents, might with advantage be studied and brought to bear throughout the town and county of Northampton. Let the committee have faith, find the right man, prepare convincing literature, enlist the aid of the press, clergy, and churches. Let the working men and women of Northampton join hands in the cause of the sick and suffering in defence of their own interests against the day of sickness. Other places have done, and are doing, this with remarkable results. Shall it be said of the most independent centre of opinion, possibly, which this country contains to-day, that when it is put to the test of practical effort and personal service, the people reveal themselves as nothing better than crumbling sand?

### THE COMMITTEE AND THE PEOPLE OF NORTHAMPTON.

At any rate, the fulfilment of the requirement of £2,000 a year additional income for this hospital is a question which must be settled between the committee of the hospital and the people of Northampton, who have so far failed adequately to realise the claims and needs of the General Hospital. Either or both of these groups are responsible for the shortage in its present income, largely, we believe, from a want of knowledge of the altered circumstances which have made modern hospitals, like that at Northampton, so increasingly costly and so increasingly helpful and indispensable to the continued health of the majority of the people. The general aspect of this hospital at present is distinctly satisfactory. It has the advantage of possessing a plain, but in many ways attractive façade facing the Billing Road, whilst the rear view of the hospital showing the new wings, when seen from the gardens at the back, gives it an air of real importance and attractiveness. It is a great thing for the patients in the verandahs, when lying in their beds, to have the noble view overlooking the gardens and the fair range of country. The people of the town and county of Northampton should be proud of their General Hospital as it exists to-day. We could wish, as we have shown in the course of our report, that they would exhibit some active consciousness of the privileges they enjoy in this connection, by displaying an earnest willingness to lend a hand, to provide the £2,000 additional income required, and deciding to take some individual part in the work of this great institution.

---

**The Harrow Cottage Hospital and the St. Albans Hospital will be reported on next week.**

